# Determination of quality markers for quality control of *Zanthoxylum nitidum* using ultra-performance liquid chromatography coupled with near infrared spectroscopy

**DOI:** 10.1371/journal.pone.0270315

**Published:** 2022-06-24

**Authors:** Xinhong Wang, Qingwen Wu, Lulu Li, Peng Wang, Yue Wang, Weifeng Wei, Xiaojun Ma, Jing Shu, Kai Zhang, Dongming Ma

**Affiliations:** 1 College of Forestry Engineering, Shandong Agriculture and Engineering University, Jinan, Shandong, China; 2 Research Center of Chinese Herbal Resource Science and Engineering, Guangzhou University of Chinese Medicine, Guangzhou, Guangdong, China; 3 Shandong Academy of Agricultural Sciences, Jinan, Shandong, China; 4 China Resources Sanjiu Medical Pharmaceutical Co, Ltd, Shenzhen, Guangdong, China; National University of Kaohsiung, TAIWAN

## Abstract

With the increasing demand for quality control in the traditional Chinese medicine industry, there is a need for the development of quality markers and a quick, non-destructive technique for the discrimination of related species. In our previous study, ultra-performance liquid chromatography (UPLC) was used for the simultaneous determination of five compounds, including three alkaloids (nitidine chloride, chelerythrine, and magnoflorine), one flavonoid (aurantiamarin), and one lignan (sesamin). In this study, the simultaneous quantification of the above-mentioned compounds could be used to discriminate the powders of roots from those of stems. To further test the reliability of the five compounds, seventy-two batches of wild and seventy-five batches of cultivated *Zanthoxylum nitidum* samples collected from Guangdong, Guangxi, and Fujian provinces in China were analyzed by UPLC and near-infrared spectroscopy (NIRS). In general, the quantitative results of UPLC were consistent with those of NIRS, and cultivated *Z*. *nitidum* has similar major bioactive compounds as the wild one, as supported by principal component analysis. Consequently, these five major bioactive compounds are suggested as potential quality markers. In addition, the NIRS method with discriminant analysis successfully differentiated *Z*. *nitidum* from three related species (*Z*. *avicennae*, *Z*. *scandens* and *Toddalia asiatica*) of the Rutaceae family. In summary, this study provides a method for the rapid identification of *Z*. *nitidum* and discrimination of root and stem powders, and suggests five compounds as quality markers for the evaluation of *Z*. *nitidum*.

## Introduction

*Zanthoxylum nitidum* (Roxb.) DC. is a widely used medicinal plant distributed in southern China, southeast Asia, and Australia [1, 2]. In the Chinese Pharmacopoeia 2020 edition, the dried roots of *Z*. *nitidum* are recorded as *Zanthoxyli Radix* and are mainly used for traumatic injury, stomachache, toothache, rheumatic arthralgia, snakebite, and burns [3]. In India, different parts of the plant are traditionally used for medicinal purposes [4]. Modern pharmacological studies have shown that *Z*. *nitidum* has anti-inflammatory [5], anti-nociceptive, and other activities [2].

**Fig 1:**
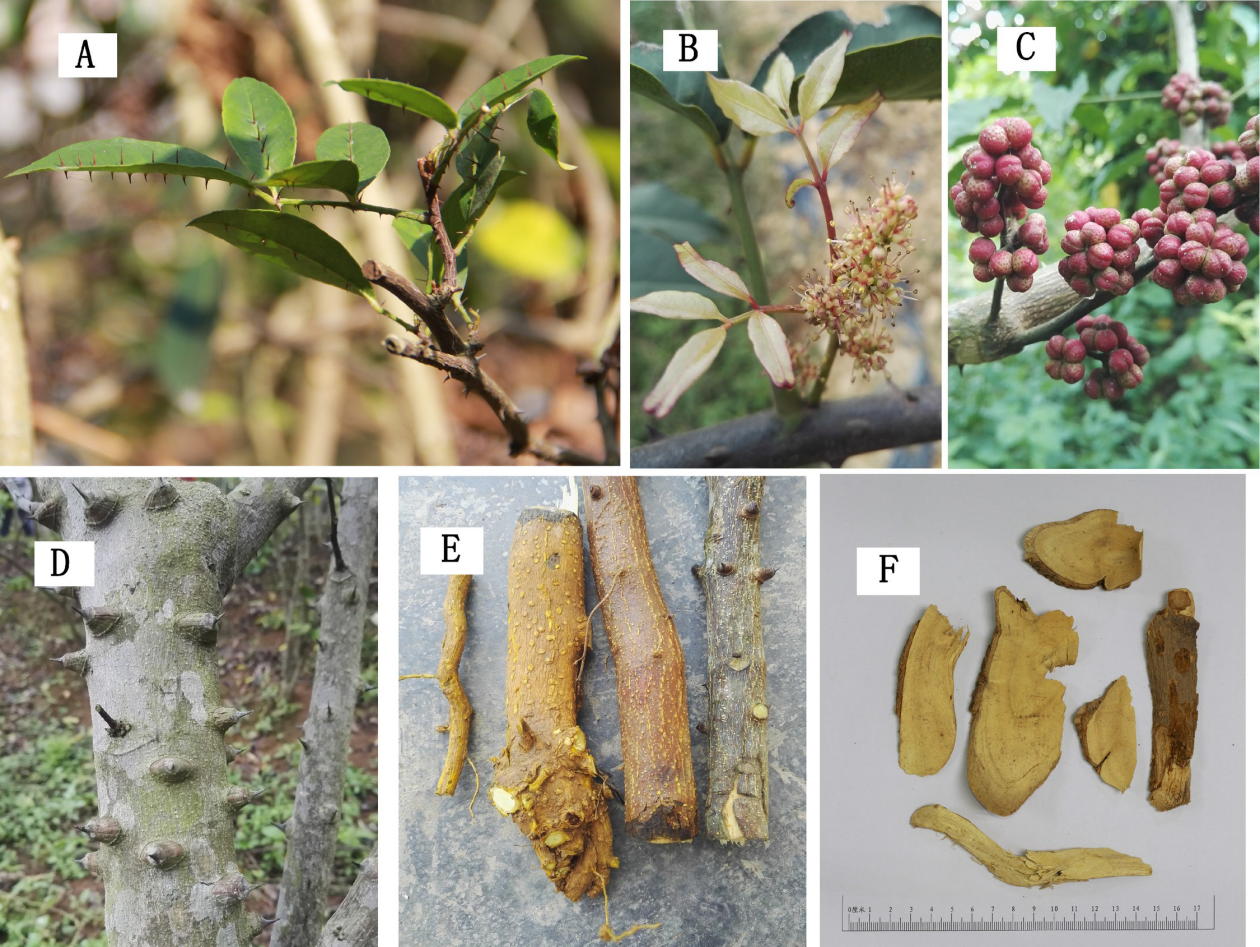
Morphology of *Zanthoxylum nitidum*. **A.** Opposite leaflet blades, two sides of leaves with prickles; **B.** Flowers 4-merous, perianth in 2 series; **C.** Fruit pedicel 2–5 mm, reddish-brown follicles; **D.** Nail-like, prickly stem; **E.** The branch root, main root, and the part connecting the root and stem from left to right; **F.** Slices of dried roots of *Z*. *nitidum*.

*Z*. *nitidum* is an evergreen woody climber with a height of 1–2 m, with an ovoid leaf that has a nail-like thorn along the vein (Fig 1A). *Z*. *nitidum* has a corymbothyrsus and flowers from March to May (Fig 1B). The fruiting period is typically from September to November (Fig 1C). Hook-like prickles are present on the stems (Fig 1D). The dried roots of *Z*. *nitidum* are harvested and widely used in Chinese medicine. The correct use of plant sources is very important for therapeutic approaches. However, the roots of *Z*. *avicennae*, *Z*. *scandens* and *Toddalia asiatica* in S1 Fig (see Supplementary material) from the Rutaceae family have appeared in herb markets, leading to confusion. This has attracted our attention for quality control of *Z*. *nitidum*.

At present, more than 150 chemical constituents have been isolated and identified from *Z*. *nitidum*, most of which include alkaloids [6], coumarins, lignans, flavonoids, terpenes, steroids, and alkylamides. There are 88 alkaloids and associated glycosides identified in *Z*. *nitidum*, and 52 benzophenanthridine alkaloids represent the most important alkaloids. Moreover, four aporphine alkaloids, four flavonoids, and fifteen lignans have been identified in *Z*. *nitidum* [6]. In our previous study, an ultra-performance liquid chromatography (UPLC) system was developed for the simultaneous quantification of the five most abundant compounds (three alkaloids, one flavonoid, and one lignan) in *Z*. *nitidum*: namely, nitidine chloride, chelerythrine, magnoline, aurantiamarin (also called hesperidin), and sesamin [7]. According to the literature, both stem and root samples contain alkaloids; however, the root has stronger anti-inflammatory activity than the stem [8]. Whether these five compounds could be used as quality markers for the discrimination of root and stem powders remains uninvestigated.

Near-infrared spectroscopy (NIRS) is currently applied in multiple industries because of its advantages, such as being rapid (some measurements are in the millisecond range), non-destructive, low-cost, and simple to use. Since the late 1980s, NIRS has been used as a continuous monitoring and process control tool for food, agriculture, and pharmaceutical industries [9, 10]. Rapid discrimination of *Flos Mume* [11], *Gentiana* [12], *Chrysanthemum* [13], *Digitalis purpurea* [14], and their related species using NIRS has been shown to be feasible. However, a method for the rapid identification of *Z*. *nitidum* and discrimination from its confused species from the same family needs to be developed.

Excessive exploitation and destruction of natural habitats have sharply reduced the natural sources of *Z*. *nitidum* [15]. Thus, *Z*. *nitidum* has been cultivated in some counties of Guangdong province to conserve *Z*. *nitidum* resources. Currently, there are no known methods and quality markers to evaluate cultivated *Z*. *nitidum*. In this study, 75 cultivated and 72 wild samples of *Z*. *nitidum* were collected and evaluated using UPLC. Then, a rapid quantitative analysis of the five major bioactive compounds was performed using NIRS.

Collectively, the three alkaloids combined with one flavonoid and one lignan can be used as potential markers for distinguishing the authenticity of *Z*. *nitidum*. In this study, quantified profiling and NIRS were employed to evaluate the quality of the roots and stems of *Z*. *nitidum*, roots of *Z*. *nitidum* and its related species, and cultivated vs wild resources using those five compounds.

## Materials and methods

### Sample preparation

Fresh root samples from 72 batches of wild and 75 batches of cultivated *Z*. *nitidum* were collected from Guangdong, Guangxi, and Fujian provinces in China (Fig 1). The roots and stems of seven cultivated *Z*. *nitidum* samples were collected from three counties in the Guangdong province. In addition, fifteen batches of *Z*. *nitidum*, five batches of *Zanthoxylum avicennae*, five batches of *Zanthoxylum scandens* and five batches of *Toddalia asiatica* root samples were collected from Guangdong and Guangxi provinces in China for discrimination and identification. All samples were authenticated by Prof. Dongming Ma and deposited at the College of Forestry Engineering of Shandong Agriculture and Engineering University. The branch root, main root, and the part connecting the root and stem are shown in Fig 1E (from left to right). Slices of dried roots of *Z*. *nitidum* are shown in Fig 1F. After air-drying in the sun, the dried samples were crushed and sifted through a 60-mesh sieve.

### NIR spectroscopic data collection

The NIR spectra of the samples were collected at 8 cm^-1^ intervals over the spectral region of 4,000–10,000 cm^-1^ using an Antaris MXFT-NIR System (Thermo Scientific, Madison, WI, USA) equipped with a hand-held optical fiber reflectance adapter. Each spectrum was obtained by averaging 64 scans. All samples were allowed to equilibrate to room temperature (25°C) before NIR spectral scanning to ensure that the samples were analyzed at the same temperature. The humidity in the laboratory was maintained at an ambient level.

### UPLC data collection

The samples were extracted with 100 mL methanol in a conical flask under heating reflux for 90 min. The extracts were then stored at 4°C. The supernatant of the extract was filtered to obtain the sample for UPLC analysis.

An improved method for the simultaneous determination of five chemical compounds using UPLC was developed in our research, which has been briefly described in reference [7]. Chromatographic analysis was performed using a Waters ACQUITY UPLC H-CLASS system. An HSS T3 column (150 mm × 2.1 mm, 1.8 μm) used for chromatographic separation was maintained at 35 ± 5°C. The mobile phase consisted of acetonitrile (A) and 0.5% aqueous formic acid (B, adjusted to a pH of 5.0, with ammonium hydroxide) and was delivered following the following gradient program: 0–1 min, linear gradient of 10–20% A; 1–5 min, 10–18% A; 5–16 min, 18–28% A; 16–27 min, 28–36% A; 27–35 min, 36–60% A; 35–40 min, 60–70% A; 40–40.1 min, 70–0% A; and 40.1–42 min, 0% A. The flow rate of the mobile phase was 0.4 mL/min with an injection volume of 2 μL. UV monitoring was performed at 273 nm.

### Data processing

TQ Analyst (version 8.0, Thermo Scientific, Madison, WI, USA) was used to divide the calibration and validation sets, mathematically pretreat the spectra, establish the calibration models, and perform other computations. Origin (version 9.1) was used to generate figures.

## Results and discussion

### UPLC analysis of five major bioactive compounds in root and stem

Nitidine chloride, a benzophenanthridine alkaloid which is the main biologically active constituent in *Z*. *nitidum*, had a root/stem content ratio ranging from 1.71 to 5.86, with a mean of 2.83 (Table 1). Chelerythrine, another benzophenanthridine alkaloid, had a larger variation of root/stem ratio than that of nitidine chloride, ranging from 1.32 to 18.5, and a mean of 3.86 (Table 1). Magnoflorine, an important quaternary aporphine alkaloid, had a moderate variation between root and stem, ranging from 1.4 to 3.21 mg/g, with a mean of 2.07 (Table 1). The content of the three alkaloids in the root was more than 2-fold of that in the stem.

**Table 1:** Five major bioactive compounds of the root and stem measured by UPLC (mg/g).

Origin	nitidine chloride			chelerythrine			magnoflorine			aurantiamarin			sesamin		
	root	stem	r/s	root	stem	r/s	root	stem	r/s	root	stem	r/s	root	stem	r/s
Dianbai, Guangdong	1.78	0.76	2.34	2.54	0.29	8.76	3.99	1.64	2.44	1.67	3.18	0.53	0.66	0.47	1.40
Gaozhou, Guangdong	0.63	0.13	4.85	0.74	0.04	18.5	4.67	1.45	3.21	1.83	1.37	1.34	0.58	0.28	2.07
Gaozhou, Guangdong	1.29	0.22	5.86	2.09	1.58	1.32	3.43	1.43	2.39	1.48	1.54	0.96	0.71	0.37	1.92
Yuntan, Guangdong	1.06	0.34	3.12	1.77	0.16	11.06	2.24	1.61	1.4	2.9	1.07	2.71	0.82	0.78	1.05
Yuntan, Guangdong	0.80	0.39	2.05	0.99	0.16	6.19	3.03	1.86	1.63	3.09	3.96	0.78	0.71	0.60	1.18
Yuntan, Guangdong	1.51	0.4	3.78	2.36	0.18	13.11	2.38	1.27	1.87	3.81	2.83	1.35	0.53	0.36	1.47
Yuntan, Guangdong	1.13	0.66	1.71	1.08	0.59	1.83	4.62	2.53	1.83	3.65	5.57	0.66	0.72	0.73	0.99
Mean (n = 7)	1.17*	0.41	2.83	1.65*	0.43	3.86	3.48*	1.68	2.07	2.63	2.79	0.94	0.68	0.51	1.32
SD	0.4	0.23	1.54	0.72	0.54	6.18	0.99	0.42	0.61	0.97	1.62	0.74	0.1	0.19	0.42

*P < 0.05, compared to the stem using Student’s *t* test.

r/s: root-to-stem content ratio. SD: standard deviation.

Interestingly, the root/stem aurantiamarin content ratio was irregular; that is, the content in some root samples was higher than that in the stems, while content in some root samples was lower than that in the stems. The mean root/stem ratio of aurantiamarin was 0.94 (Table 1). Sesamin was more abundant in most root samples than in the stems; one exception was the sample from Yuntan, Guangdong, where the root/stem ratio was close to 1. The mean root/stem ratio of sesamin was 1.32 (Table 1). Principal component analysis (PCA) was performed to obtain a global view of the four metabolite profile differentiations in samples from the two tissues, and the results showed a separation between root and stem samples (S2 Fig). Collectively, a comprehensive comparison of the above four major bioactive compounds, excluding aurantiamarin, can discriminate root and stem samples.

*Z*. *nitidum* is a perennial medicinal plant harvested throughout the year, with the overground parts removed after 5–6 years of growth. The underground roots are used as the medicinal part based on the Pharmacopoeia of the People’s Republic of China [6]. In a study on the determination of nitidine chloride in different parts of *Z*. *nitidum*, it was found that the root contained 0.15% nitidine chloride and the old branch contained 0.06% [16]. The fact that the content in roots is 2.5-fold that in stems is comparable with the mean root/stem content ratio (2.8) in our study (Table 1). It is noticeable that some root samples from Gaozhou and Yuntan have comparable content to that of the stem. Thus, if only nitidine chloride is used as an indicator, it is difficult to discriminate between root and stem slices or powders. Both roots and stems of *Z*. *nitidum* have reported anti-inflammatory activity, with roots having stronger activity than stems [17]. Moreover, polar extracts of *Z*. *nitidum* roots and stems have different antibacterial activities [18]. To prevent the misuse of roots and stems, nitidine chloride coupled with two other alkaloids and one lignan is suggested as a quality marker for the correct use of *Z*. *nitidum*.

### Application of NIR spectroscopy for the discrimination of *Z*. *nitidum* from its confused species

NIR spectroscopy is a rapid, powerful, non-destructive, and low-cost alternative with significant practical advantages compared to conventional methods of analysis [19–21]. Herein, NIR spectroscopy was used to rapidly distinguish *Z*. *nitidum* from its related species.

Discriminant analysis, distance matching, and quality control (QC) compare search can be used to identify the spectrum of an unknown sample by comparing it to multiple types of standard samples. Discriminant analysis was used in this study. The optimum selection of wavebands for the calibration models of identification was 4,119.21–9,881.46 cm^-1^. The mahalanobis distances to the four species were obtained from the computational simulations by TQ Analyst software using raw spectrum with no pre-treatment (Table 2). Minimum mahalanobis distance was selected as the predicted species. The rate of consistency of identification by detecting classical taxonomy morphology was 100%, indicating very good reliability.

**Table 2:** Validation of the results of mahalanobis distance in the false identification model.

No.	Mahalanobis distance				Predicted species	True or False
	Distance to *Z*. *nitidum*	Distance to *Z*. *avicennae*	Distance to *Z*. *scandens*	Distance to *T*. *asiatica*		
1	3.3462	5.2485	5.6797	5.5248	*Z*. *nitidum*	True
2	0.6925	1.7914	2.2175	1.7464	*Z*. *nitidum*	True
3	0.7370	1.7700	2.4403	2.1017	*Z*. *nitidum*	True
4	1.0561	2.5509	3.1498	2.8673	*Z*. *nitidum*	True
5	2.0518	2.6290	2.2601	2.2123	*Z*. *nitidum*	True
6	1.2334	2.0760	2.1563	1.5464	*Z*. *nitidum*	True
7	1.5442	2.4957	2.3390	2.1846	*Z*. *nitidum*	True
8	0.8631	1.9857	2.5159	1.9774	*Z*. *nitidum*	True
9	1.7711	2.8211	3.1405	2.6548	*Z*. *nitidum*	True
10	1.4613	3.2957	3.7207	3.6664	*Z*. *nitidum*	True
11	1.4194	1.9444	2.2396	2.2904	*Z*. *nitidum*	True
12	1.5750	2.8132	3.1744	2.9642	*Z*. *nitidum*	True
13	0.9634	1.5240	2.2830	2.0205	*Z*. *nitidum*	True
14	1.0252	2.7622	3.1876	2.6012	*Z*. *nitidum*	True
15	1.0922	2.9623	3.2503	2.6864	*Z*. *nitidum*	True
16	2.1897	0.1278	1.0214	1.2258	*Z*. *avicennae*	True
17	2.2515	0.1261	0.9847	1.2582	*Z*. *avicennae*	True
18	2.0943	0.2228	1.1771	1.4398	*Z*. *avicennae*	True
19	2.2045	0.1329	1.1638	1.4237	*Z*. *avicennae*	True
20	2.1946	0.0352	1.0593	1.2845	*Z*. *avicennae*	True
21	2.6312	1.0672	0.0910	1.0188	*Z*. *scandens*	True
22	2.7854	1.1624	0.2034	1.0819	*Z*. *scandens*	True
23	3.0363	1.5039	0.6589	1.0552	*Z*. *scandens*	True
24	2.6871	1.1646	0.1166	1.0056	*Z*. *scandens*	True
25	2.5875	1.0181	0.1596	1.1162	*Z*. *scandens*	True
26	2.4980	1.4389	1.0959	0.1860	*T*. *asiatica*	True
27	2.2745	1.2361	1.0167	0.1906	*T*. *asiatica*	True
28	2.6660	1.5706	1.1532	0.3706	*T*. *asiatica*	True
29	2.2884	1.3185	1.0725	0.1224	*T*. *asiatica*	True
30	2.4557	1.3961	1.0655	0.1425	*T*. *asiatica*	True

Mahalanobis distances to the four species were determined using the discriminant analysis method. The predicted species were determined according to the minimum mahalanobis distance. The "True or False" column shows the distinguished results between the real and predicted species.

Some plants of the Rutaceae family have been reported to contain nitidine chloride. For example, *Zanthoxylum rubescens* contains nitidine chloride and chelerythrine [22]. Magnoflorine and nitidine chloride have been detected in *Zanthoxylum scandens* [23]. *Toddalia asiatica* from the Rutaceae family contains nitidine chloride, magnoflorine and chelerythrine [24]. However, unlike *Z*. *nitidum*, *T*. *asiatica* does not contain sesamin. Except for the qualitative chemical properties, the quantitative concentrations of the five major bioactive compounds among *Z*. *nitidum* and three confused species were also distinctive. This might make NIRS suitable for a rapid and cost-effective method for identifying *Z*. *nitidum*. NIRS has the potential to differentiate *Z*. *nitidum*, *Z*. *avicennae*, *Z*. *scandens* and *Toddalia asiatica* from the Rutaceae family.

### Quality markers tested in cultivated and wild *Z*. *nitidum* using UPLC

**Fig 2:**
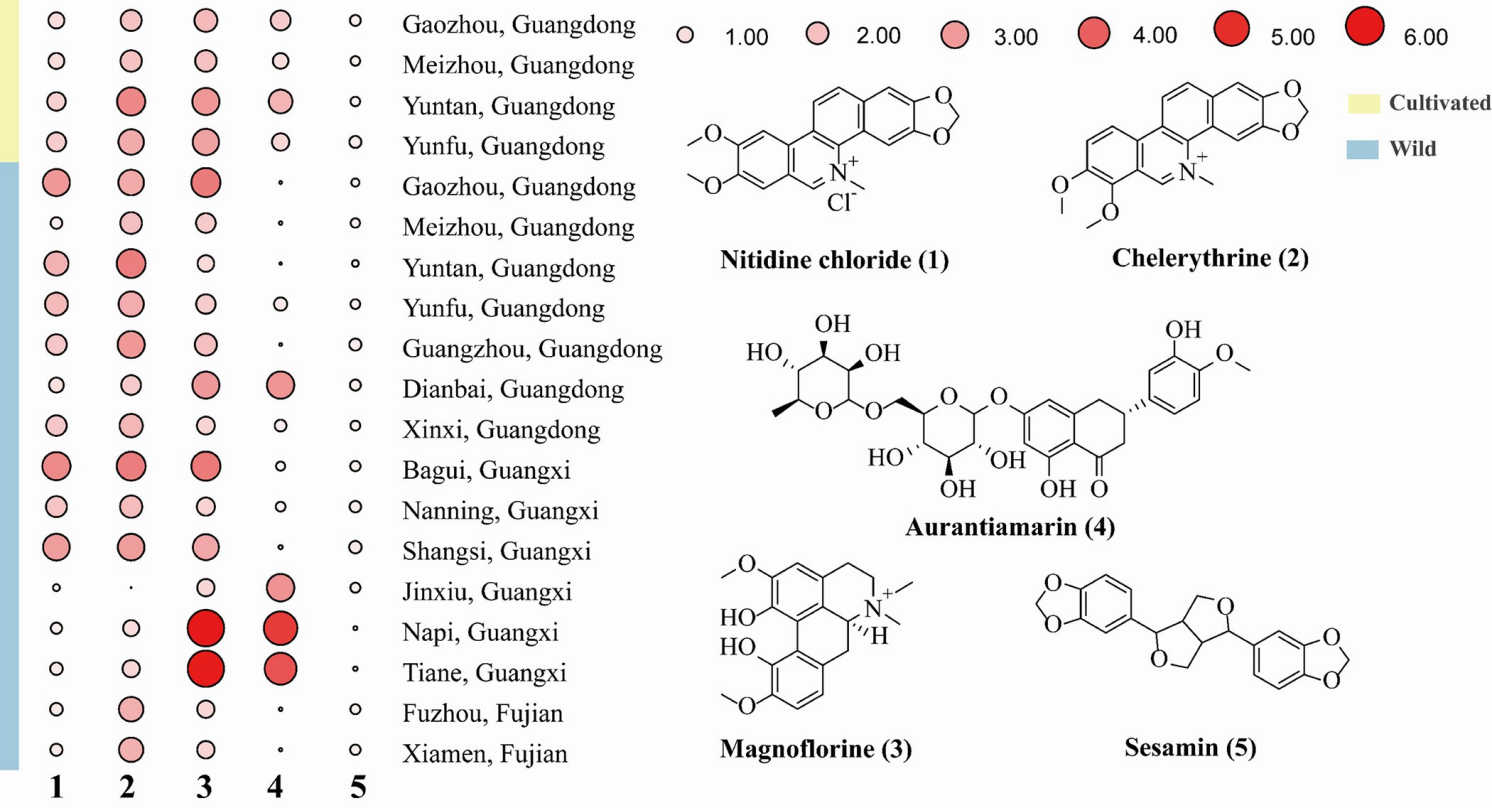
Heat map showing the contents of five active components in *Z*. *nitidum* from different regions.

**Fig 3:**
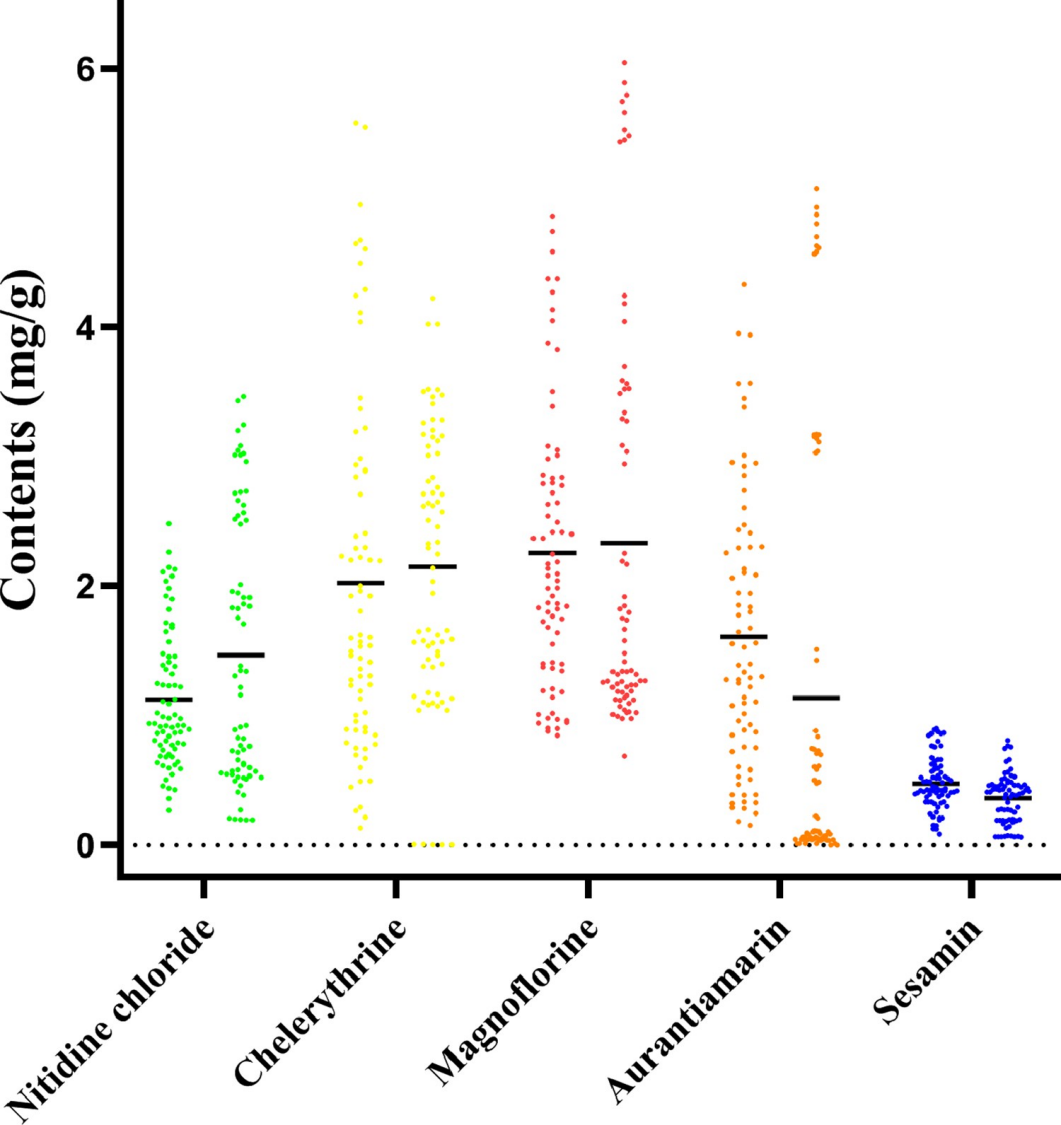
Distribution of five constituents of *Z*. *nitidum*. The right corresponds to wild samples, while the left corresponds to cultivated samples. The black horizontal lines indicate the mean values of different groups.

The range of nitidine chloride concentration of cultivated plants from 4 counties in Guangdong Province was 0.27 to 2.48 mg/g (Fig 2 and Table 3). In fact, these four cultivated species were the counterparts of wild plants from the same county. For wild *Z*. *nitidum*, except for 4 counties in Guangdong province, plants from three counties in Guangdong province, six counties in Guangxi province and two counties in Fujian province were harvested and subjected to UPLC analysis. The concentration of nitidine chloride from wild plants from these 15 counties ranged from 0.19 to 3.47 mg/g. Chelerythrine had a more varied concentration in a larger range than that of nitidine chloride. The mean concentration of chelerythrine in the cultivated and wild plants was 80% and 50% higher than that of nitidine chloride, respectively. The highest chelerythrine content was found in cultivated plants from Yuntan, Guangdong, up to 5.58 mg/g (Figs 2 and 3, Table 3). Magnoflorine had a comparable amount in cultivated and wild plants with that of chelerythrine; in contrast, the highest magnoflorine content was found in wild plants from Tiane, Guangxi, up to 6.05 mg/g (Figs 2 and 3, Table 3).

**Table 3:** Chemical composition of cultivated and wild *Z*. *nitidum* measured by UPLC (mg/g).

Statistic	nitidine chloride (mg/g)		chelerythrine (mg/g)		magnoflorine (mg/g)		aurantiamarin(mg/g)		sesamin(mg/g)	
	cultivated(n = 75)	wild (n = 72)	cultivated (n = 75)	wild (n = 72)	cultivated (n = 75)	wild (n = 72)	cultivated (n = 75)	wild (n = 72)	cultivated (n = 75)	wild (n = 72)
minimum	0.2682	0.1936	0.1322	0.0033	0.8470	0.6885	0.1532	0	0.0858	0.0622
maximum	2.4819	3.4671	5.5769	4.2251	4.8597	6.0478	4.3300	5.0731	0.9000	0.8087
mean	1.1240	1.4658	2.0249	2.1483	2.2592	2.3359	1.6098	1.1358	0.4764	0.3641

The mean content of aurantiamarin in cultivated plants was higher than that in wild plants; conversely, the mean content of the three above-mentioned alkaloids in wild plants was higher than that in cultivated plants (Figs 2 and 3, Table 3). Of five major bioactive compounds, the mean concentration of sesamin was the lowest, up to 0.48 and 0.36 mg/g in cultivated and wild plants, respectively (Table 3).

In general, the content of five major bioactive compounds in cultivated *Z*. *nitidum* was comparable with wild samples (Figs 2 and 3, Table 3), as supported by PCA analysis S3A Fig (see Supplementary material). The first two PCs [PC1 (39.0%) versus PC2 (30.3%)] and PC3 (19.2%) were distributed in a disorderly manner, where wild and cultivated samples did not cluster separately. These results, coupled with the discrimination of roots and stems with these five ingredients, suggest that nitidine chloride, chelerythrine, magnoflorine, aurantiamarin, and sesamin can act as markers for the quality control of *Z*. *nitidum*.

### Spectral features and establishment and spectral pre-treatment selection for the calibration models

**Fig 4:**
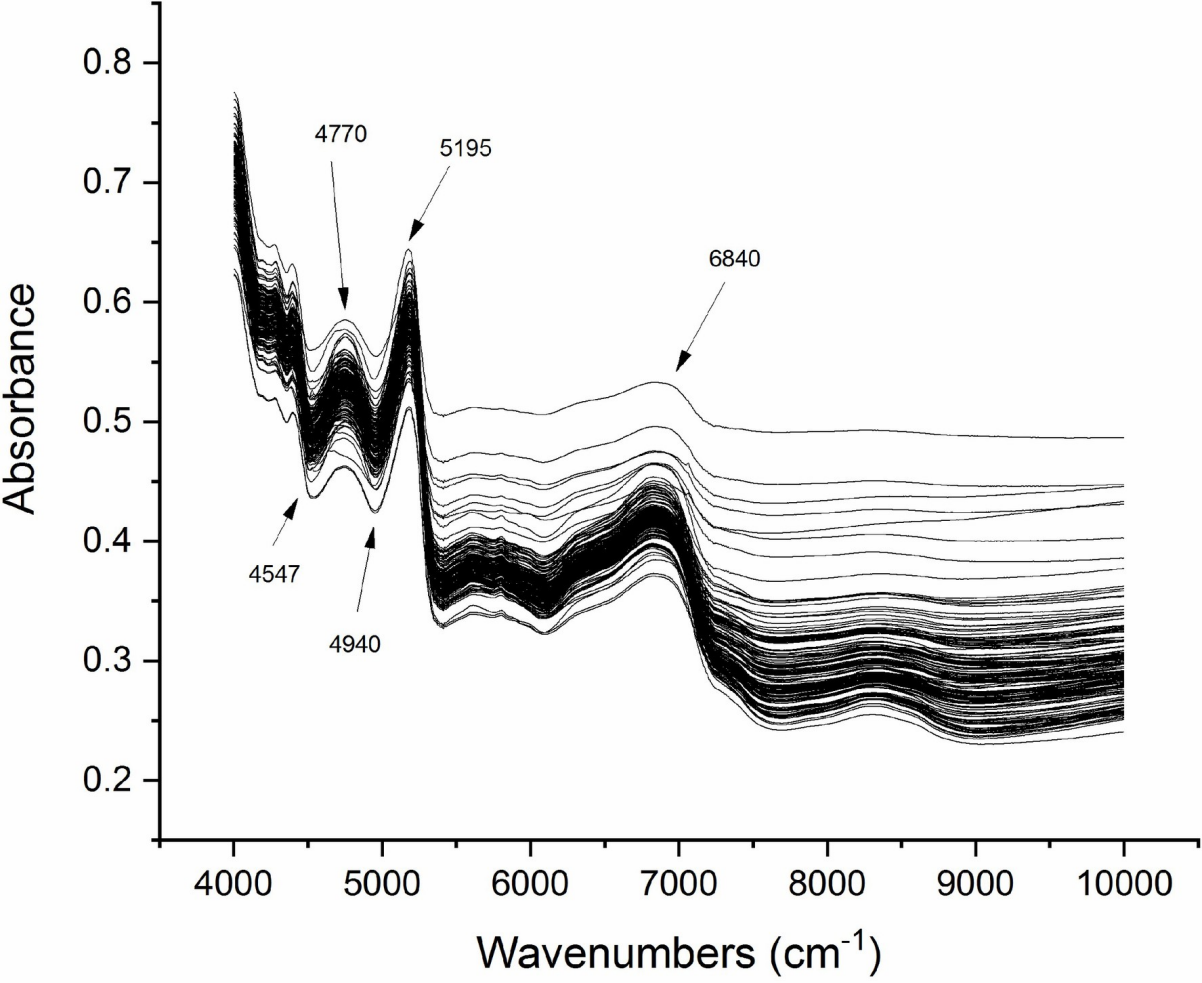
Near infrared spectra of the *Z*. *nitidum* samples. The arrows indicate the stretching vibration of hydrogen-containing functional groups (O-H, N-H, C-H).

Here, NIR spectroscopy was examined as a possible alternative to UPLC for the analysis of five major bioactive compounds in *Z*. *nitidum* root powder. The spectra of the sample set of *Z*. *nitidum* plants are shown in Fig 4. Partial least squares regression (PLSR) is a classic modeling method that has been widely applied in quantitative models because of the high quality of its results. The advantages of PLSR include its good forecasting ability and relative simplicity. PLSR has also been widely applied to establish quantitative calibration models for traditional Chinese medicine [25]. PLSR explains the maximum amount of variability in the data by reducing the dimensionality of the spectral data by calculating the factors. Based on the pre-treated NIR spectra, an NIR quantitative analysis model for the five bioactive components in *Z*. *nitidum* was established using the PLSR with UPLC analysis data as the true values. The 147 samples were randomly divided into calibration and validation sets at a 3:1 ratio. The accuracy of the calibration models is influenced by light scattering, which introduces non-linearities that can significantly influence the spectra. Both are processes in which electromagnetic radiation is scattered. This problem is commonly resolved by preprocessing the spectra prior to the modelling step. The application of a scatter correction method to in-line processing of NIR spectra is ubiquitous; no exception has been found in the literature. In most cases, the choice of preprocessing method is empirical or arbitrary. The three most used scatter correction methods are multiplicative scatter correction (MSC), standard normal variate (SNV), and spectral derivatives [26, 27]. In this study, MSC and SNV were tested to reduce the effects of particle size.

Table 4 shows the spectral pre-treatment data and its results for the calibration models. The effects of SNV and MSC on the correlation coefficient of determination were optimized. Derivatives can remove both additive and multiplicative effects in the spectra and have been used in analytical spectroscopy for decades [26]. As shown in Table 4, the spectral derivatives have a significant impact on the R^2^ and root mean square error of calibration (RMSEC) of the calibration models. To achieve the optimal predicted effect, spectral pre-treatment methods with the highest R^2^ and lowest RMSEC were chosen for the calibration models. MSC and the first derivative (FD) are considered effective options for nitidine chloride, chelerythrine, magnoflorine, and aurantiamarin. For the desired effect, the spectra were smoothed using the Savitzky Golay (SG) filter algorithm before derivation to prevent noise magnification. By comparing R^2^, MSC-SD-SG was applied to build a calibration model for sesamin in *Z*. *nitidum*.

**Table 4:** The correlation coefficient (R^2^) and the root mean square error of calibration (RMSEC) of spectral pre-treatment methods for the calibration models.

Compounds	Method	R^2^	RMSEC	Method	R^2^	RMSEC
nitidine chloride	MSC-Spectrum	0.8395	0.442	SNV-Spectrum	0.8340	0.428
	MSC-FD	0.9394	0.266	SNV-FD	0.9394	0.266
	MSC-SD	0.8991	0.340	SNV-SD	0.9001	0.338
chelerythrine	MSC-Spectrum	0.9276	0.445	SNV-Spectrum	0.9225	0.460
	MSC-FD	0.9707	0.286	SNV-FD	0.9707	0.286
	MSC-SD	0.9464	0.385	SNV-SD	0.9464	0.385
magnoflorine	MSC-Spectrum	0.7214	0.762	SNV-Spectrum	0.7127	0.772
	MSC-FD	0.9573	0.318	SNV-FD	0.9540	0.321
	MSC-SD	0.9075	0.462	SNV-SD	0.9023	0.475
aurantiamarin	MSC-Spectrum	0.7923	0.738	SNV-Spectrum	0.7893	0.743
	MSC-FD	0.9423	0.405	SNV-FD	0.9422	0.405
	MSC-SD-SG	0.9180	0.357	SNV-SD-SG	0.9182	0.479
sesamin	MSC-Spectrum	0.7486	0.127	SNV-Spectrum	0.7908	0.117
	MSC-FD	0.8346	0.077	SNV-FD	0.9272	0.072
	MSC-SD-SG	0.9418	0.064	SNV-SD-ND	0.8182	0.110

MSC, multiplicative scatter correction; SNV, standard normal variate; FD, first derivative; SD, second derivative; SG, Savitzky Golay smoothing with a polynomial order of 3 fitted over a 5-nm interval.

### Selection of the optimum number of factors for the calibration models

**Fig 5:**
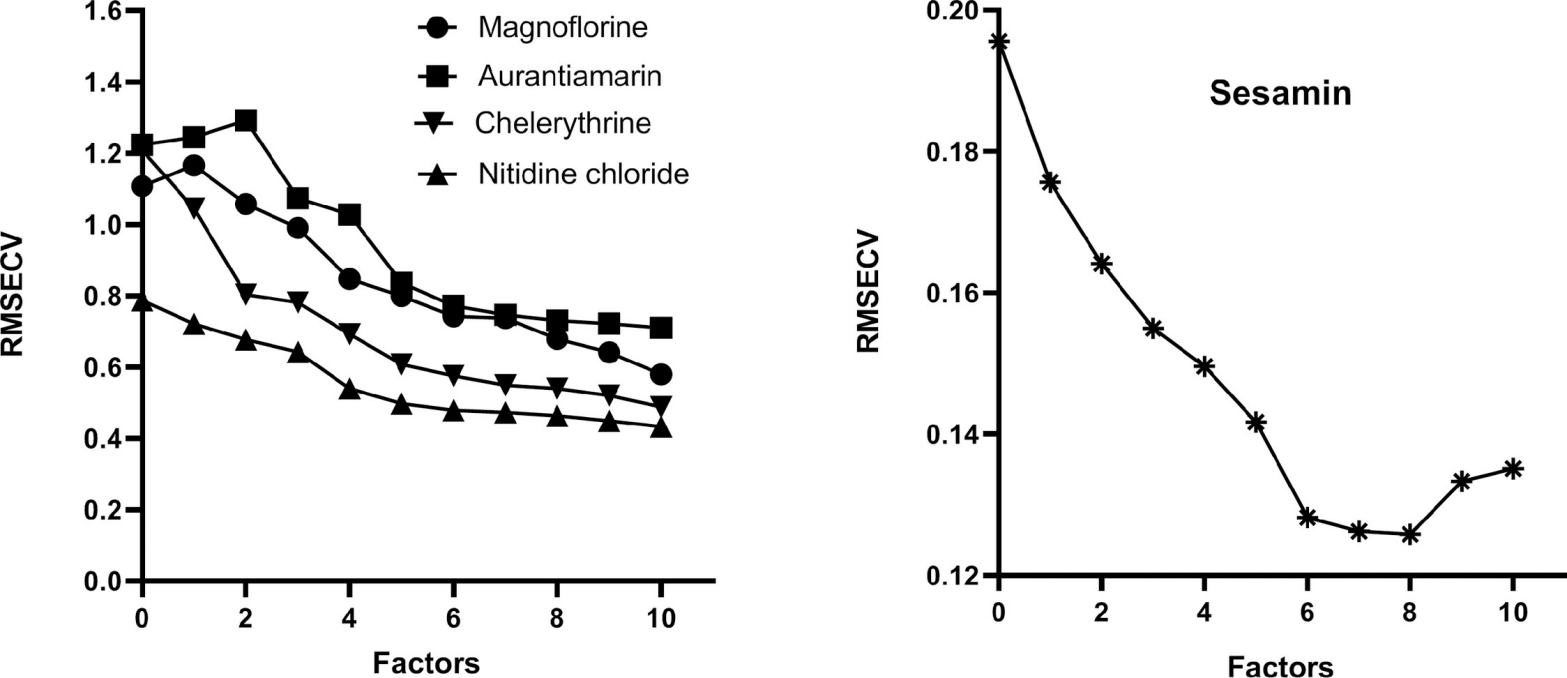
Effect of different principal factors on RMSECV of the calibration set. **A.** nitidine chloride, chelerythrine, magnoflorine and aurantiamarin; **B.** sesamin.

The "underfittedness" problem is caused by insufficient information resulting from a limited number of factors; however, choosing more factors than the optimum values introduced in the model will bring about the "overfittedness" problem. Either "underfittedness" or "overfittedness" will reduce the predictive power of the established models [28]. The optimum number of factors corresponding to the lowest RMSECV values was selected for the calibration models. The relationship between RMSECV and the factors for all five compounds is shown in Fig 5. Therefore, the optimum selection of factors for the calibration model of sesamin was eight, and the remaining four components were 10.

### Selection of the wave band for the calibration models

The spectral band between 4,000 and 10,000 cm^-1^ has the advantages of abundant information and high intensity [29]. The software-optimized spectrum band is often intercepted for analysis because it avoids unnecessary interference effects of additional peaks. In many cases, the optimized band of the software cannot contain all the information of the compound to be simulated; therefore, RMSECV and Rc^2^ are used to select the optimal band. Lower RMSECV and higher Rc^2^ values indicate more reasonable band selection. According to RMSECV and Rc^2^ shown in Table 5, the optimum wave bands for the calibration models of nitidine chloride, chelerythrine, magnoflorine, aurantiamarin, and sesamin were 4,200–4,600 cm^-1^, 4,200–5,400 cm^-1^, 4,000–6,000 cm^-1^, 4,300–5,300 cm^-1^, and 4,300–5,800 cm^-1^, respectively.

**Table 5:** Calibration and validation results for quality parameters of *Z*. *nitidum* using PLS models.

Compound	Wave Band cm^-1^	Pre-treatment spectral	Calibration set		Cross-validation		Validation set		RPD
			RMSEC	Rc^2^	RMSECV	Rcv^2^	RMSEV	Rv^2^	
nitidine chloride	4200–4600	MSC-FD	0.266	0.9394	0.433	0.8353	0.279	0.9075	2.1878
chelerythrine	4200–5400	MSC-FD	0.286	0.9707	0.488	0.9137	0.449	0.9391	2.7565
magnoflorine	4000–6000	MSC-FD	0.318	0.9573	0.58	0.8536	0.5	0.9559	3.1062
aurantiamarin	4300–5300	MSC-FD	0.405	0.9423	0.711	0.8173	0.758	0.9148	2.0405
sesamin	4300–5800	MSC-SD-SG	0.0642	0.9418	0.126	0.756	0.0542	0.8509	1.7291

RMSEC: root mean square error of calibration, Rc^2^: correlation coefficient of determination in calibration, RMSECV: root mean square error of cross-validation, Rcv^2^: correlation coefficient of determination in cross-validation, RMSEV: root mean square error of validation, Rv^2^: correlation coefficient of determination in validation, and RPD: ratio of validation to deviation.

### Evaluation of the established models

**Fig 6:**
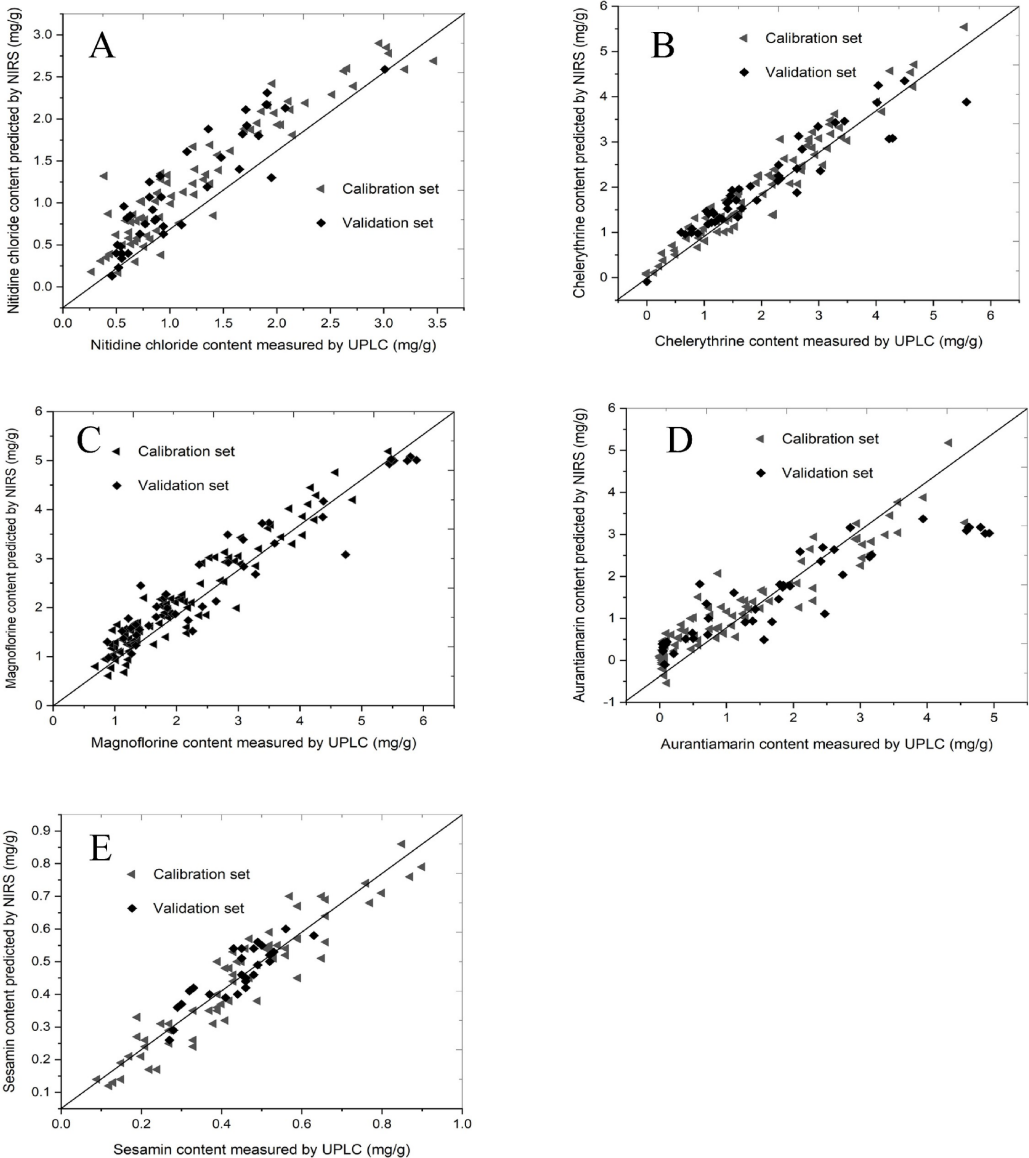
Scatter plots of measured and predicted values for the abundance of five constituents of *Z*. *nitidum* in the calibration and validation sets: **A.** nitidine chloride; **B.** chelerythrine; **C.** magnoflorine; **D.** aurantiamarin; **E.** sesamin. ◀ represents calibration ◆ represents validation; The straight lines show the regressive curve with the equation y = x.

The calibration and predictive ability of the model were evaluated using R^2^, RMSEC, RMSECV, and RMSEV [30], and the ratio of validation to deviation (RPD) was used as the standard for evaluation of the established models [31]. The model was also cross-validated using UPLC (Fig 6).

The dataset of all 147 samples was divided into calibration and validation sets in a ratio of approximately 3 to 1 using the kennard-stone method. Based on the data treatment procedure and the largest absolute regression coefficient, the best predictive performance PLS models for each quality parameter were built, as shown in Table 5. The RMSEC values for the calibration models of nitidine chloride, chelerythrine, magnoflorine, aurantiamarin, and sesamin were 0.2660, 0.2860, 0.3180, 0.4050, and 0.0642, respectively. The correlation coefficient of determination in calibration (Rc^2^) values between 0.94 and 0.97 of five compounds. The correlation coefficient of determination in validation (Rv^2^) values of nitidine chloride, chelerythrine, magnoflorine, and aurantiamarin were greater than 0.90, the Rv^2^ value of sesamin was 0.85. The RPD is defined as the ratio of the standard deviation of the response variable to RMSECV. The RPD values of chelerythrine and magnoflorinen were greater than 2.5 and 3, respectively, indicating good and excellent prediction accuracy. The RPD values of nitidine chloride and aurantiamarin were between 2.0 and 2.5, indicating that semi-quantitative validations or rough estimations are possible. The RPD value of sesamin was 1.72, indicating that the model can discriminate low from high values of the response variable [32]. PCA analysis using NIRS-based analysis of five major bioactive compounds showed no difference with UPLC in the wild and cultivated samples (S3 Fig).

The accuracy of NIRS methods depends on the particle size, homogeneity, temperature, and presentation of the sample. The key to this problem is the choice of chemometric methods and spectral pre-treatment. Various chemometrics methods, such as PCA and PLS, have been employed extensively. Spectral pre-treatments, such as smoothing, derivative methods, MSC, SNV, centering methods, and normalization, aim to remove variations in spectra due to changes in the laboratory environment and sample.

## Conclusions

Quantitative applications for the analysis of natural products have been reported. For alkaloids, there have been reported methods for the determination of three main xanthine alkaloids in roasted coffee, caffeine, theobromine and theophylline using PLS models based on NIR spectroscopy and HPLC [33]. In addition, isoquinoline alkaloids, such as berberine, jatrorrhizine, and palmatine, were analyzed simultaneously by NIR spectroscopy and HPLC to differentiate species of *Phellodendri cortex*, *Phellodendri chinense cortex* and *Phellodendri amurensis cortex* [34]. Similarly, NIR spectroscopy was used to determine the berberine, palmatine, jatrorrhizine, and total alkaloid contents of coptis extracts [35]. However, methodologies for benzophenanthridine and aporphine-type alkaloids have not been investigated. In this study, the first application of NIRS to simultaneously analyze nitidine chloride, chelerythrine, and magnoflorine in *Z*. *nitidum* was reported, with good predictive results.

Five major bioactive compounds in *Z*. *nitidun* were simultaneously determined using NIRS. After spectral pre-treatments, PLS was utilized to build calibration models. The results suggest that each model was reliable. We verified the feasibility of the simultaneous determination of multiple compounds, particularly trace constituents, in a short time using NIRS. Collectively, quality control of *Z*. *nitidum* using five major compounds by UPLC coupled with NIRS has the potential to discriminate different tissue powders (e.g., root and stem) of *Z*. *nitidum* and make a quick distinction between *Z*. *nitidum* and its related species.

## Supporting information

S1 Fig Root morphological variation among *Z*. *nitidum* and its related species. The right represents *Z*. *nitidum*, while the left represents *T*. *asiatica*.
(DOCX)

S2 Fig Principal Component Analysis for the four metabolites from root and stem tissues. The black circles represent root samples, while the red ones represent stem samples.
(DOCX)

S3 Fig PCA score 3D plot of cultivated and wild *Zanthoxylum nitidum* samples using ultra-high performance liquid chromatography (A) and near infrared spectroscopy (B). The black circles represent cultivated samples, while the red ones represent wild samples. 147 samples from different growth regions were used as contributes for PCA (S1 Table). The blue number represents the highest content of five major representative compounds from 147 samples detailed in S1 Table.
(DOCX)

S1 Table Habitats, types and the bioactive compound content of 147 *Z*. *nitudum* samples using ultra-high performance liquid chromatography and near infrared spectroscopy. (DOCX)

S2 Table Calibration curves of the five major bioactive constituents of *Z*. *nitidum*. (DOCX)
