# Supplementary material for: Determination of quality markers for quality control of Zanthoxylum nitidum using ultra-performance liquid chromatography coupled with near infrared spectroscopy
Source: PLoS One. 2022 Jun 24;17(6):e0270315. doi: 10.1371/journal.pone.0270315 (PMC9231700; doi:10.1371/journal.pone.0270315)
Supplement: S1 Fig — The right represents Z. nitidum, while the left represents T. asiatica. (DOCX) [file pone.0270315.s001.docx]

#
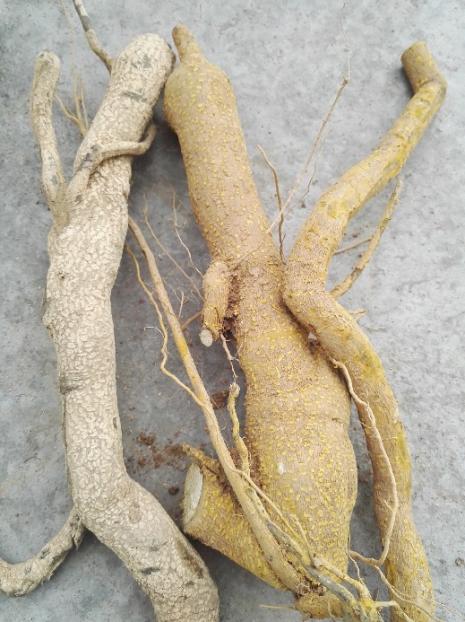


**S1 Fig. Root morphological variation among *Z. nitidum* and its related species.** The right represents *Z. nitidum*, while the left represents *T. asiatica.*
