# Supplementary material for: Determination of quality markers for quality control of Zanthoxylum nitidum using ultra-performance liquid chromatography coupled with near infrared spectroscopy
Source: PLoS One. 2022 Jun 24;17(6):e0270315. doi: 10.1371/journal.pone.0270315 (PMC9231700; doi:10.1371/journal.pone.0270315)
Supplement: S2 Fig — The black circles represent root samples, while the red ones represent stem samples. (DOCX) [file pone.0270315.s002.docx]

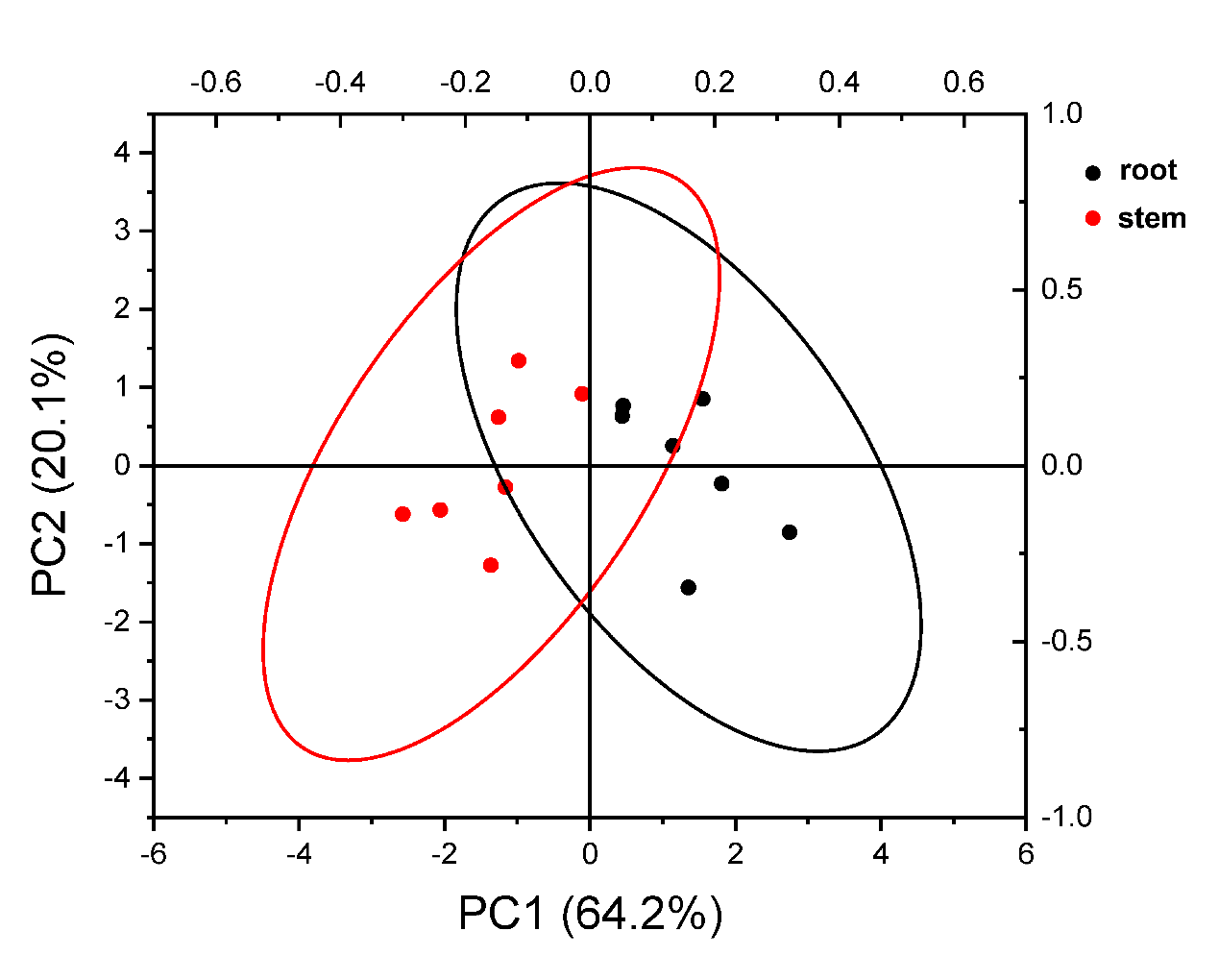


**S2 Fig. Principal Component Analysis for the four metabolites from root and stem tissues.** The black circles represent root samples, while the red ones represent stem samples.
