# Supplementary material for: Determination of quality markers for quality control of Zanthoxylum nitidum using ultra-performance liquid chromatography coupled with near infrared spectroscopy
Source: PLoS One. 2022 Jun 24;17(6):e0270315. doi: 10.1371/journal.pone.0270315 (PMC9231700; doi:10.1371/journal.pone.0270315)
Supplement: S3 Fig — PCA score 3D plot of cultivated and wild Zanthoxylum nitidum samples using ultra-high performance liquid chromatography (A) and near infrared spectroscopy (B). The black circles represent cultivated samples, while the red ones represent wild samples. 147 samples from different growth regions were used as contributes for PCA (S1 Table). The blue number represents the highest content of five major representative compounds from 147 samples detailed in S1 Table. (DOCX) [file pone.0270315.s003.docx]

**S3 Fig. PCA score 3D plot of cultivated and wild *Zanthoxylum nitidum* samples using ultra-high performance liquid chromatography (A) and near infrared spectroscopy (B).** The black circles represent cultivated samples, while the red ones represent wild samples. 147 samples from different growth regions were used as contributes for PCA (S1 Table). The blue number represents the highest content of five major representative compounds from 147 samples detailed in S1 Table
