# Supplementary material for: Determination of quality markers for quality control of Zanthoxylum nitidum using ultra-performance liquid chromatography coupled with near infrared spectroscopy
Source: PLoS One. 2022 Jun 24;17(6):e0270315. doi: 10.1371/journal.pone.0270315 (PMC9231700; doi:10.1371/journal.pone.0270315)
Supplement: S1 Table — (DOCX) [file pone.0270315.s004.docx]

**S1 Table. Habitats, types and the bioactive compound content of 147 *Z. nitudum* samples using ultra-high performance liquid chromatography and near infrared spectroscopy.**

| No. | Type | Habitat | Content（mg/g） | | | | | | | | | |
| --- | --- | --- | --- | --- | --- | --- | --- | --- | --- | --- | --- | --- |
|  |  |  | magnoflorine | | aurantiamarin | | nitidine chloride | | chelerythrine | | sesamin | |
|  |  |  | UPLC | NIR | UPLC | NIR | UPLC | NIR | UPLC | NIR | UPLC | NIR |
| 1 | cultivated | Gaozhou, Guangdong | 2.04 | 2.19 | 3.95 | 3.88 | 1.25 | 1.21 | 2.94 | 2.72 | 0.43 | 0.54 |
| 2 | cultivated | Gaozhou, Guangdong | 2.49 | 1.85 | 0.89 | 0.79 | 0.43 | 0.87 | 0.45 | 0.71 | 0.33 | 0.24 |
| 3 | cultivated | Gaozhou, Guangdong | 2.64 | 2.13 | 1.11 | 1.61 | 0.81 | 1.25 | 0.78 | 0.99 | 0.37 | 0.19 |
| 4 | cultivated | Gaozhou, Guangdong | 1.99 | 2.11 | 1.28 | 1.42 | 0.88 | 0.67 | 0.96 | 1 | 0.24 | 0.17 |
| 5 | cultivated | Gaozhou, Guangdong | 1.73 | 1.68 | 0.24 | 0.37 | 0.92 | 1.35 | 1.31 | 1.35 | 0.45 | 0.12 |
| 6 | cultivated | Gaozhou, Guangdong | 0.97 | 1.54 | 1.65 | 1.41 | 0.74 | 0.81 | 1.24 | 1.01 | 0.42 | 0.17 |
| 7 | cultivated | Gaozhou, Guangdong | 1.40 | 1.54 | 0.75 | 0.75 | 0.87 | 1.02 | 1.23 | 1.24 | 0.43 | 0.1 |
| 8 | cultivated | Gaozhou, Guangdong | 1.77 | 1.84 | 0.93 | 1.27 | 1.22 | 1.23 | 1.50 | 1.38 | 0.32 | 0.21 |
| 9 | cultivated | Gaozhou, Guangdong | 1.18 | 1.47 | 0.53 | 0.59 | 0.59 | 0.79 | 0.27 | 0.54 | 0.15 | 0.14 |
| 10 | cultivated | Meizhou, Guangdong | 2.79 | 3.13 | 0.96 | 0.64 | 1.70 | 1.88 | 2.89 | 2.87 | 0.52 | 0.59 |
| 11 | cultivated | Meizhou, Guangdong | 1.21 | 1.25 | 0.87 | -1.21 | 0.60 | 2.52 | 2.39 | 1.9 | 0.41 | -0.61 |
| 12 | cultivated | Meizhou, Guangdong | 2.72 | 2.55 | 1.01 | 1.16 | 1.24 | 1.4 | 0.89 | 0.67 | 0.25 | 0.31 |
| 13 | cultivated | Meizhou, Guangdong | 1.64 | 1.25 | 0.47 | 1 | 0.92 | 0.38 | 0.13 | 0.11 | 0.15 | 0.19 |
| 14 | cultivated | Meizhou, Guangdong | 1.14 | 1.34 | 0.18 | 0.61 | 0.68 | 0.3 | 0.22 | 0.25 | 0.21 | 0.24 |
| 15 | cultivated | Meizhou, Guangdong | 2.40 | 2.49 | 0.85 | 0.53 | 0.98 | 1.33 | 1.54 | 1.41 | 0.38 | 0.31 |
| 16 | cultivated | Meizhou, Guangdong | 2.54 | 3.02 | 3.57 | 3.77 | 1.32 | 1.28 | 2.20 | 2.06 | 0.77 | 0.68 |
| 17 | cultivated | Meizhou, Guangdong | 0.85 | 0.95 | 0.15 | -4.46 | 0.99 | **7.37** | 4.95 | 4.25 | 0.33 | -0.37 |
| 18 | cultivated | Yuntan, Guangdong | 2.80 | 2.93 | 1.30 | 1.28 | 1.13 | 1.13 | 1.54 | 1.13 | 0.37 | 0.35 |
| 19 | cultivated | Yuntan, Guangdong | 4.37 | 3.85 | 3.94 | 3.37 | 2.08 | 2.13 | **5.58** | 3.88 | 0.33 | 0.35 |
| 20 | cultivated | Yuntan, Guangdong | 4.58 | 4.76 | 3.45 | 3.45 | 2.13 | 2.11 | 5.55 | **5.54** | 0.42 | 0.8 |
| 21 | cultivated | Yuntan, Guangdong | 1.38 | 1.69 | 0.76 | 1.08 | 0.91 | 0.83 | 0.70 | 0.87 | 0.32 | 0.41 |
| 22 | cultivated | Yuntan, Guangdong | 2.42 | 2.91 | 2.09 | 1.26 | 0.92 | 1.32 | 1.96 | 2.24 | 0.40 | 0.37 |
| 23 | cultivated | Yuntan, Guangdong | 2.83 | 3.49 | 2.47 | 1.11 | 1.36 | 1.88 | 4.50 | 4.35 | 0.42 | 0.38 |
| 24 | cultivated | Yunfu, Guangdong | 4.27 | 4.29 | 1.14 | 0.56 | 2.27 | 2.19 | 4.67 | 4.71 | 0.54 | 0.55 |
| 25 | cultivated | Yunfu, Guangdong | 2.86 | 3.02 | 2.31 | 2.94 | 1.82 | 1.95 | 3.22 | 3.18 | 0.89 | 0.63 |
| 26 | cultivated | Yunfu, Guangdong | 2.17 | 1.6 | 0.39 | 0.69 | 1.38 | 1.69 | 1.13 | 1.56 | 0.39 | 0.36 |
| 27 | cultivated | Yunfu, Guangdong | 3.01 | 3.05 | 2.06 | 1.84 | 1.01 | 0.99 | 1.46 | 1.05 | 0.76 | 0.74 |
| 28 | cultivated | Yunfu, Guangdong | 2.10 | 2.26 | 0.33 | 0.85 | 1.23 | 1.1 | 2.90 | 3.22 | 0.39 | 0.35 |
| 29 | cultivated | Gaozhou, Guangdong | 0.90 | 0.61 | 0.32 | 1.57 | 0.78 | 2.54 | 2.00 | 0.4 | 0.86 | 0.78 |
| 30 | cultivated | Gaozhou, Guangdong | 2.37 | 1.84 | 0.39 | 0.52 | 1.46 | 1.39 | 1.00 | 1.09 | 0.53 | 0.53 |
| 31 | cultivated | Gaozhou, Guangdong | 3.88 | 3.3 | 0.58 | 0.48 | 2.04 | 1.93 | 3.20 | 3.39 | 0.80 | 0.71 |
| 32 | cultivated | Gaozhou, Guangdong | 2.25 | 2.1 | 0.33 | 0.73 | 1.39 | 2.62 | 3.37 | 3.32 | 0.65 | 0.7 |
| 33 | cultivated | Gaozhou, Guangdong | 3.05 | 3.43 | 0.51 | 1.02 | 1.45 | 1.57 | 2.84 | 3.07 | 0.66 | 0.56 |
| 34 | cultivated | Gaozhou, Guangdong | 3.50 | 3.73 | 0.60 | 1.82 | 1.90 | 2.17 | 4.04 | 4.25 | 0.52 | 0.55 |
| 35 | cultivated | Gaozhou, Guangdong | 4.14 | 4.11 | 0.58 | 1.51 | 2.11 | 2.21 | 4.61 | 4.54 | 0.44 | 0.5 |
| 36 | cultivated | Gaozhou, Guangdong | 3.83 | 4.02 | 3.57 | 3.04 | 1.92 | 2.16 | 4.65 | 4.22 | 0.88 | 0.49 |
| 37 | cultivated | Gaozhou, Guangdong | 4.05 | 3.86 | 3.01 | 2.26 | 1.98 | 2.07 | 4.25 | 4.57 | 0.87 | 0.76 |
| 38 | cultivated | Gaozhou, Guangdong | 4.38 | 4.17 | 2.74 | 2.04 | 1.72 | 1.92 | 3.45 | 3.46 | 0.59 | 0.67 |
| 39 | cultivated | Gaozhou, Guangdong | 4.86 | 4.2 | 2.30 | 1.42 | 2.15 | 1.81 | 4.11 | 3.67 | 0.57 | 0.7 |
| 40 | cultivated | Gaozhou, Guangdong | 3.08 | 3.39 | 1.39 | 0.94 | 1.65 | 1.4 | 2.71 | 2.84 | 0.43 | 0.65 |
| 41 | cultivated | Gaozhou, Guangdong | 3.39 | 3.72 | 1.68 | 0.92 | 1.68 | 1.82 | 2.99 | 3.34 | 0.66 | 0.64 |
| 42 | cultivated | Gaozhou, Guangdong | 2.84 | 2.92 | 1.28 | 0.91 | 1.48 | 1.54 | 2.30 | 2.49 | 0.59 | 0.57 |
| 43 | cultivated | Gaozhou, Guangdong | 2.14 | 2.12 | 1.25 | 1.11 | 1.23 | 1.74 | 2.20 | 2.21 | 0.85 | **0.86** |
| 44 | cultivated | Gaozhou, Guangdong | 2.63 | 3.03 | 1.08 | 0.83 | 1.57 | 1.62 | 2.41 | 2.63 | 0.86 | 0.52 |
| 45 | cultivated | Gaozhou, Guangdong | 4.74 | 3.08 | 1.56 | 0.49 | 2.48 | 1.47 | 4.29 | **3.08** | 0.45 | 0.51 |
| 46 | cultivated | Gaozhou, Guangdong | 2.78 | 2.53 | 2.26 | 2.65 | 1.02 | 1.08 | 2.23 | 2.39 | 0.90 | 0.79 |
| 47 | cultivated | Gaozhou, Guangdong | 1.75 | 2.04 | 2.96 | 2.91 | 0.50 | 0.62 | 1.36 | 1.01 | 0.41 | 0.32 |
| 48 | cultivated | Gaozhou, Guangdong | 1.01 | 1.19 | 0.28 | 0.53 | 0.64 | 0.77 | 0.29 | 0.38 | 0.13 | 0.13 |
| 49 | cultivated | Gaozhou, Guangdong | 2.19 | 2 | 1.34 | 1.22 | 1.09 | 0.76 | 2.22 | 1.4 | 0.12 | 0.12 |
| 50 | cultivated | Gaozhou, Guangdong | 1.87 | 1.8 | 2.93 | 2.89 | 0.68 | 0.54 | 1.62 | 1.92 | 0.39 | 0.5 |
| 51 | cultivated | Gaozhou, Guangdong | 2.37 | 2.88 | 2.85 | 3.16 | 0.84 | 0.92 | 2.29 | 2.14 | 0.42 | 0.48 |
| 52 | cultivated | Gaozhou, Guangdong | 2.08 | 2.16 | 2.95 | 3.26 | 0.73 | 0.82 | 1.92 | 2.1 | 0.41 | 0.57 |
| 53 | cultivated | Gaozhou, Guangdong | 1.82 | 1.9 | 2.13 | 2.36 | 0.69 | 0.78 | 1.44 | 1.31 | 0.29 | 0.36 |
| 54 | cultivated | Gaozhou, Guangdong | 1.80 | 2.15 | 0.29 | 0.55 | 0.98 | 1.24 | 1.60 | 1.86 | 0.22 | 0.17 |
| 55 | cultivated | Gaozhou, Guangdong | 1.87 | 1.97 | 1.22 | 1.44 | 0.89 | 1.11 | 1.57 | 1.76 | 0.41 | 0.39 |
| 56 | cultivated | Gaozhou, Guangdong | 1.93 | 1.88 | 2.10 | 2.59 | 0.81 | 1.07 | 1.61 | 1.96 | 0.51 | 0.54 |
| 57 | cultivated | Gaozhou, Guangdong | 1.83 | 1.4 | 1.29 | 1.01 | 0.44 | 0.39 | 1.02 | 0.81 | 0.21 | 0.26 |
| 58 | cultivated | Gaozhou, Guangdong | 1.98 | 1.87 | 0.72 | 0.61 | 0.92 | 1.07 | 1.92 | 1.71 | 0.19 | 0.27 |
| 59 | cultivated | Gaozhou, Guangdong | 1.55 | 1.63 | 1.78 | 1.46 | 1.11 | 0.74 | 1.27 | 1.32 | 0.77 | 0.53 |
| 60 | cultivated | Gaozhou, Guangdong | 0.95 | 0.77 | 3.38 | 2.99 | 0.36 | 0.31 | 0.85 | 1.32 | 0.56 | 0.52 |
| 61 | cultivated | Gaozhou, Guangdong | 1.02 | 1.29 | 4.33 | **5.18** | 0.27 | -0.47 | 0.75 | 1.08 | 0.49 | 0.49 |
| 62 | cultivated | Gaozhou, Guangdong | 1.40 | 1.55 | 0.39 | 0.51 | 0.77 | 0.75 | 1.19 | 1.23 | 0.09 | 0.14 |
| 63 | cultivated | Gaozhou, Guangdong | 1.37 | 1.34 | 1.10 | 1.06 | 0.77 | 0.76 | 0.50 | 0.51 | 0.33 | 0.26 |
| 64 | cultivated | Gaozhou, Guangdong | 2.42 | 2.02 | 2.44 | 2.69 | 0.86 | 0.79 | 1.81 | 2.02 | 0.49 | 0.56 |
| 65 | cultivated | Gaozhou, Guangdong | 1.35 | 1.3 | 1.53 | 1.67 | 0.62 | 0.58 | 0.87 | 0.98 | 0.30 | 0.37 |
| 66 | cultivated | Gaozhou, Guangdong | 0.98 | 1 | 2.61 | 2.64 | 0.46 | 0.13 | 0.79 | 1.08 | 0.43 | 0.53 |
| 67 | cultivated | Gaozhou, Guangdong | 0.96 | 1.17 | 1.95 | 1.83 | 0.64 | 0.51 | 0.74 | 1.12 | 0.50 | 0.55 |
| 68 | cultivated | Gaozhou, Guangdong | 0.88 | 1.3 | 1.84 | 1.74 | 0.61 | 0.4 | 0.60 | 1 | 0.48 | 0.54 |
| 69 | cultivated | Gaozhou, Guangdong | 0.90 | 0.98 | 2.41 | 2.36 | 0.55 | 0.34 | 0.67 | 0.96 | 0.49 | 0.49 |
| 70 | cultivated | Gaozhou, Guangdong | 1.19 | 1.56 | 1.95 | 1.77 | 0.94 | 0.72 | 0.89 | 0.98 | 0.68 | 0.56 |
| 71 | cultivated | Gaozhou, Guangdong | 0.94 | 1.25 | 1.40 | 1.4 | 0.79 | 0.61 | 0.49 | 0.6 | 0.52 | 0.5 |
| 72 | cultivated | Gaozhou, Guangdong | 2.98 | 1.99 | 2.30 | 1.72 | 1.41 | 0.85 | 2.20 | 1.38 | 0.52 | 0.54 |
| 73 | cultivated | Gaozhou, Guangdong | 1.41 | 1.52 | 1.56 | 1.63 | 0.70 | 0.58 | 0.91 | 0.88 | 0.61 | 0.41 |
| 74 | cultivated | Gaozhou, Guangdong | 1.85 | 1.99 | 1.85 | 1.79 | 0.94 | 0.63 | 1.41 | 1.52 | 0.63 | 0.58 |
| 75 | cultivated | Gaozhou, Guangdong | 1.68 | 2.02 | 1.80 | 1.81 | 0.87 | 0.81 | 1.31 | 1.3 | 0.62 | 0.44 |
| 76 | wild | Guangzhou, Guangdong | 3.34 | 3.2 | 0.07 | 0.09 | 2.72 | 2.39 | 2.84 | 3.02 | 0.39 | 0.4 |
| 77 | wild | Guangzhou, Guangdong | 0.69 | 0.8 | 0.00 | -0.71 | 0.83 | 0.86 | 3.26 | 3.45 | 0.81 | 0.41 |
| 78 | wild | Meizhou, Guangdong | 1.58 | 1.62 | 0.04 | -0.05 | 0.39 | 1.32 | 2.64 | 2.07 | 0.77 | 0.57 |
| 79 | wild | Meizhou, Guangdong | 0.98 | 1.04 | 0.05 | 0.22 | 0.77 | 0.8 | 2.25 | 2.4 | 0.39 | 0.36 |
| 80 | wild | Meizhou, Guangdong | 4.24 | 3.79 | 0.06 | 0.18 | 0.56 | 0.5 | 1.10 | 1.22 | 0.14 | 0.49 |
| 81 | wild | Meizhou, Guangdong | 1.16 | 0.68 | 0.07 | -0.36 | 0.27 | 0.18 | 1.38 | 1.26 | 0.20 | 0.21 |
| 82 | wild | Meizhou, Guangdong | 1.05 | 1.65 | 0.00 | 0.1 | 0.82 | 0.57 | 2.04 | 1.85 | 0.27 | 0.31 |
| 83 | wild | Meizhou, Guangdong | 1.67 | 1.81 | 0.04 | 0.39 | 0.46 | 1.31 | 3.03 | 2.36 | 0.66 | 0.69 |
| 84 | wild | Meizhou, Guangdong | 0.98 | 0.98 | 0.05 | 0.46 | 0.76 | 0.48 | 2.14 | 2.27 | 0.29 | 0.41 |
| 85 | wild | Meizhou, Guangdong | 1.19 | 0.83 | 0.11 | -0.54 | 0.41 | 0.35 | 1.10 | 1.44 | 0.17 | 0.21 |
| 86 | wild | Fuzhou, Fujian | 1.14 | 1.37 | 0.06 | 0.41 | 0.72 | 0.63 | 2.33 | 2.19 | 0.33 | 0.42 |
| 87 | wild | Fuzhou, Fujian | 1.24 | 1.36 | 0.04 | 0.25 | 0.66 | 0.86 | 2.57 | 2.6 | 0.45 | 0.54 |
| 88 | wild | Fuzhou, Fujian | 1.34 | 1.65 | 0.05 | 0.31 | 0.60 | 0.82 | 2.62 | 1.88 | 0.46 | 0.54 |
| 89 | wild | Xiamen, Fujian | 1.35 | 1.44 | 0.05 | 0.23 | 0.57 | 0.96 | 2.62 | 2.41 | 0.52 | 0.52 |
| 90 | wild | Xiamen, Fujian | 1.12 | 1.52 | 0.05 | 0.22 | 0.63 | 0.85 | 2.30 | 2.25 | 0.37 | 0.4 |
| 91 | wild | Xiamen, Fujian | 1.32 | 1.33 | 0.04 | 0 | 0.62 | 0.65 | 2.51 | 2.08 | 0.45 | 0.46 |
| 92 | wild | Heyuan, Guangdong | 1.18 | 1.48 | 0.04 | 0.19 | 0.58 | 1.11 | 2.46 | 2.24 | 0.46 | 0.44 |
| 93 | wild | Heyuan, Guangdong | 1.26 | 1.24 | 0.04 | -0.09 | 0.73 | 1.02 | 3.08 | 2.49 | 0.46 | 0.45 |
| 94 | wild | Heyuan, Guangdong | 1.32 | 1.27 | 0.04 | -0.2 | 0.74 | 1.01 | 3.16 | 2.86 | 0.48 | 0.46 |
| 95 | wild | Napi, Guangxi | 5.79 | 5.08 | 4.87 | 3.02 | 0.55 | 0.39 | 1.15 | 1.45 | 0.06 | 0.5 |
| 96 | wild | Napi, Guangxi | 5.44 | **5.19** | 4.57 | 3.28 | 0.52 | 0.17 | 1.04 | 1.32 | 0.07 | 0.51 |
| 97 | wild | Napi, Guangxi | 5.45 | 4.93 | 4.59 | 3.09 | 0.50 | 0.4 | 1.04 | 1.47 | 0.06 | 0.51 |
| 98 | wild | Tiane, Guangxi | 4.18 | 4.45 | 0.88 | 2.07 | 1.34 | 1.34 | 1.95 | 2.26 | 0.13 | 0.5 |
| 99 | wild | Tiane, Guangxi | 5.52 | 5 | 4.62 | 3.17 | 0.52 | 0.23 | 1.07 | 1.18 | 0.06 | 0.52 |
| 100 | wild | Tiane, Guangxi | 5.89 | 5.01 | 4.93 | 3.03 | 0.55 | 0.4 | 1.17 | 1.41 | 0.07 | 0.48 |
| 101 | wild | Tiane, Guangxi | 5.48 | 5.03 | 4.63 | 3.17 | 0.51 | 0.5 | 1.08 | 1.41 | 0.06 | 0.5 |
| 102 | wild | Tiane, Guangxi | **6.05** | 5 | **5.07** | 3.1 | 0.57 | 0.33 | 1.18 | 1.27 | 0.07 | 0.47 |
| 103 | wild | Tiane, Guangxi | 5.74 | 5 | 4.80 | 3.17 | 0.54 | 0.48 | 1.13 | 1.22 | 0.07 | 0.45 |
| 104 | wild | Tiane, Guangxi | 5.66 | 4.99 | 4.70 | 3.14 | 0.53 | 0.41 | 1.10 | 1.3 | 0.07 | 0.51 |
| 105 | wild | Dianbai, Guangdong | 2.95 | 2.95 | 3.03 | 2.44 | 0.89 | 1.12 | 1.54 | 1.42 | 0.49 | 0.38 |
| 106 | wild | Dianbai, Guangdong | 3.04 | 2.89 | 3.16 | 2.53 | 0.92 | 1.28 | 1.62 | 1.4 | 0.50 | 0.34 |
| 107 | wild | Dianbai, Guangdong | 3.09 | 2.84 | 3.14 | 2.46 | 0.91 | 1.32 | 1.66 | 1.53 | 0.51 | 0.32 |
| 108 | wild | Xizhen, Guangdong | 2.26 | 1.52 | 0.50 | 0.52 | 1.95 | 1.3 | 1.43 | 1.69 | 0.45 | 0.5 |
| 109 | wild | Xizhen, Guangdong | 2.17 | 1.48 | 0.48 | 0.27 | 1.83 | 1.82 | 1.38 | 1.71 | 0.43 | 0.46 |
| 110 | wild | Xizhen, Guangdong | 2.19 | 1.74 | 0.49 | 0.65 | 1.83 | 1.8 | 1.40 | 1.65 | 0.43 | 0.56 |
| 111 | wild | Gaozhou, Guangdong | 3.49 | 3.62 | 0.04 | 0.06 | 3.03 | 2.85 | 2.72 | 2.48 | 0.27 | 0.29 |
| 112 | wild | Gaozhou, Guangdong | 3.53 | 3.69 | 0.04 | 0.13 | 3.05 | 2.78 | 2.71 | 2.37 | 0.27 | 0.25 |
| 113 | wild | Gaozhou, Guangdong | 3.57 | 3.76 | 0.04 | 0.16 | 3.09 | 2.73 | 2.76 | 2.38 | 0.28 | 0.29 |
| 114 | wild | Gaozhou, Guangdong | 3.53 | 3.82 | 0.03 | 0.07 | 3.01 | 2.57 | 2.71 | 2.31 | 0.27 | 0.26 |
| 115 | wild | Yuntan, Guangdong | 1.02 | 0.93 | 0.01 | 0.06 | 2.52 | 2.29 | 3.50 | 3.03 | 0.19 | 0.33 |
| 116 | wild | Yuntan, Guangdong | 1.03 | 1.26 | 0.01 | -0.07 | 2.48 | 2.56 | 3.52 | 2.89 | 0.18 | 0.32 |
| 117 | wild | Yuntan, Guangdong | 1.02 | 1.07 | 0.01 | -0.1 | 2.55 | 2.52 | 3.46 | 2.93 | 0.19 | 0.33 |
| 118 | wild | Yuntan, Guangdong | 1.01 | 0.98 | 0.01 | -0.1 | 2.57 | 2.43 | 3.52 | 2.89 | 0.19 | 0.34 |
| 119 | wild | Yuntan, Guangdong | 0.99 | 1.03 | 0.01 | -0.13 | 2.51 | 2.58 | 3.48 | 3.06 | 0.20 | 0.37 |
| 120 | wild | Yuntan, Guangdong | 1.75 | 2.17 | 0.10 | 0.64 | 1.86 | 2.09 | 3.21 | 3.48 | 0.19 | 0.64 |
| 121 | wild | Yunfu, Guangdong | 1.80 | 2.2 | 0.11 | 0.44 | 1.91 | 2.17 | 3.29 | 3.43 | 0.20 | 0.6 |
| 122 | wild | Yunfu, Guangdong | 1.74 | 2.04 | 0.10 | 0.47 | 1.84 | 1.99 | 3.17 | 3.33 | 0.19 | 0.62 |
| 123 | wild | Yunfu, Guangdong | 1.92 | 2.18 | 1.51 | 1.24 | 2.01 | 1.93 | 1.65 | 1.54 | 0.53 | 0.51 |
| 124 | wild | Yunfu, Guangdong | 1.83 | 2.27 | 1.43 | 1.22 | 1.91 | 2.31 | 1.56 | 1.71 | 0.56 | 0.6 |
| 125 | wild | Yunfu, Guangdong | 1.07 | 1.1 | 0.58 | 0.36 | 2.63 | 2.57 | 3.01 | 3.08 | 0.47 | 0.57 |
| 126 | wild | Yunfu, Guangdong | 1.12 | 1.08 | 0.61 | 0.65 | 2.73 | 2.63 | 3.15 | 3.02 | 0.39 | 0.55 |
| 127 | wild | Xinxi, Guangdong | 1.12 | 1.12 | 0.61 | 0.49 | 2.73 | 2.7 | 3.12 | 3.05 | 0.38 | 0.56 |
| 128 | wild | Xinxi, Guangdong | 1.27 | 1.53 | 0.75 | 1.02 | 1.38 | 1.23 | 1.66 | 1.66 | 0.47 | 0.45 |
| 129 | wild | Xinxi, Guangdong | 1.19 | 1.76 | 0.71 | 1.11 | 1.31 | 1.5 | 1.57 | 1.52 | 0.44 | 0.4 |
| 130 | wild | Xinxi, Guangdong | 1.22 | 1.78 | 0.73 | 1 | 1.35 | 1.19 | 1.59 | 1.34 | 0.46 | 0.42 |
| 131 | wild | Xinxi, Guangdong | 1.85 | 2.07 | 0.11 | 0.48 | 1.96 | 2.42 | 3.29 | 3.62 | 0.25 | 0.61 |
| 132 | wild | Bagui, Guangxi | 3.29 | 2.85 | 0.09 | 0.35 | **3.47** | 2.69 | 4.02 | 3.89 | 0.43 | 0.46 |
| 133 | wild | Bagui, Guangxi | 3.28 | 2.68 | 0.09 | 0.38 | 3.43 | 2.57 | 4.02 | 3.87 | 0.45 | 0.54 |
| 134 | wild | Bagui, Guangxi | 4.05 | 3.48 | 0.84 | 0.76 | 2.96 | 2.9 | 2.34 | 3.06 | 0.43 | 0.44 |
| 135 | wild | Nanning, Guangxi | 1.42 | 2.45 | 0.70 | 1.35 | 1.71 | 2.11 | 1.46 | 1.81 | 0.37 | 0.52 |
| 136 | wild | Nanning, Guangxi | 1.48 | 2.2 | 0.74 | 1.25 | 1.76 | 1.87 | 1.58 | 1.78 | 0.41 | 0.48 |
| 137 | wild | Nanning, Guangxi | 1.26 | 1.58 | 0.21 | 0.16 | 1.16 | 1.61 | 1.49 | 1.93 | 0.65 | 0.51 |
| 138 | wild | Nanning, Guangxi | 1.34 | 1.68 | 0.22 | 0.22 | 1.22 | 1.67 | 1.57 | 1.76 | 0.75 | 0.4 |
| 139 | wild | Nanning, Guangxi | 1.34 | 1.23 | 0.08 | -0.1 | 3.25 | 2.27 | 4.23 | 3.07 | 0.54 | 0.36 |
| 140 | wild | Shangsi, Guangxi | 1.10 | 1.09 | 0.06 | -0.2 | 2.66 | 2.6 | 3.41 | 3.1 | 0.59 | 0.45 |
| 141 | wild | Shangsi, Guangxi | 3.70 | 3.44 | 0.08 | 0.06 | 3.20 | 2.59 | 2.81 | 2.92 | 0.56 | 0.54 |
| 142 | wild | Shangsi, Guangxi | 3.59 | 3.31 | 0.07 | -0.09 | 3.01 | 2.59 | 2.65 | 3.13 | 0.76 | 0.52 |
| 143 | wild | Jinxiu, Guangxi | 1.27 | 1.06 | 3.17 | 2.51 | 0.20 | -0.41 | 0.00 | -0.09 | 0.41 | 0.67 |
| 144 | wild | Jinxiu, Guangxi | 1.24 | 1.11 | 3.17 | 2.83 | 0.19 | -0.3 | 0.00 | 0.07 | 0.41 | 0.71 |
| 145 | wild | Jinxiu, Guangxi | 1.27 | 1 | 3.17 | 2.75 | 0.20 | -0.47 | 0.00 | -0.02 | 0.42 | 0.67 |
| 146 | wild | Jinxiu, Guangxi | 1.22 | 0.94 | 3.04 | 2.76 | 0.20 | -0.22 | 0.00 | 0.1 | 0.41 | 0.64 |
| 147 | wild | Jinxiu, Guangxi | 1.25 | 0.94 | 3.11 | 2.7 | 0.21 | -0.34 | 0.01 | 0.07 | 0.42 | 0.64 |
