# Supplementary material for: Determination of quality markers for quality control of Zanthoxylum nitidum using ultra-performance liquid chromatography coupled with near infrared spectroscopy
Source: PLoS One. 2022 Jun 24;17(6):e0270315. doi: 10.1371/journal.pone.0270315 (PMC9231700; doi:10.1371/journal.pone.0270315)
Supplement: S2 Table — (DOCX) [file pone.0270315.s005.docx]

**S2 Table. Calibration curves of the five major bioactive constituents of *Z. nitidum.***

| compound | Regression equation | Linear range  （μg/mL） | R^2^ | LOD | LOQ |
| --- | --- | --- | --- | --- | --- |
| nitidine chloride | Y=27456800X - 4429.3 | 1.88～47 | 0.9999 | 0.095 | 0.377 |
| chelerythrine | Y=29299100X - 18340 | 8.12～203 | 0.9990 | 0.090 | 0.404 |
| magnoflorine | Y=6244400X - 4437.8 | 7.96～199 | 0.9999 | 0.160 | 0.396 |
| aurantiamarin | Y=6232390X + 6965.5 | 8.04～201 | 0.9998 | 0.089 | 0.399 |
| sesamin | Y=3371840X + 1584.2 | 2.52～63 | 0.9999 | 0.050 | 0.255 |

Y: peak area; X: concentration of compound (μg/mL); LOD: limit of detection, S / N = 3; LOQ: limit of quantification, S / N = 10.
